# A Method of Optimizing Characteristic Impedance Compensation Using Cut-Outs in High-Density PCB Designs

**DOI:** 10.3390/s22030964

**Published:** 2022-01-26

**Authors:** Vaidotas Barzdenas, Aleksandr Vasjanov

**Affiliations:** Department of Computer Science and Communications Technologies, Vilnius Gediminas Technical University, 03227 Vilnius, Lithuania; aleksandr.vasjanov@vilniustech.lt

**Keywords:** compensation, cut-out, DGS, discontinuity, high-density, high-speed, impedance, optimization, PCB

## Abstract

The modern era of technology contains a myriad of high-speed standards and proprietary serial digital protocols, which evolve alongside the microwave and RF realm. The increasing data rate push the requirements for hardware design, including modern printed circuit boards (PCB). One of these requirements for modern high-speed PCB interfaces are a homogenous track impedance all the way from the source to the load. Even though some high-speed interfaces don’t require any external components embedded into the interconnects, there are others which require either passive or active components—or both. Usually, component package land-pads are of fixed size, thus, if not addressed, they create discontinuities and degrade the transmitted signal. To solve this problem, impedance compensation techniques such as reference plane cut-out are employed for multiple case studies covering this topic. This paper presents an original method of finding the optimal cut-out size for the maximum characteristic impedance compensation in high-density multilayer PCB designs, which has been verified via theoretical estimation, computer simulation, and practical measurement results. Track-to-discontinuity ratios of 1:1.75, 1:2.5, and 1:5.0 were selected in order to resemble most practical design scenarios on a 6-layer standard thickness PCB. The measurements and simulations revealed that the compensated impedance saturation occurs at (150–250%) cut-out widths for a 50 Ω microstrip.

## 1. Introduction

A growing number of high-speed standards and proprietary serial protocols are posing major design layout challenges for modern printed circuit board (PCB) designers. These serial standards include Universal Serial Bus [1], PCIe Gen1 and PCIe Gen2, Gbps Ethernet [2], LVDS [3], Serial RapidIO^®^ (SRIO) [4], Common Public Radio Interface (CPRI) [5], Double Data Rate (DDR) [6], OBSAI, SD/HD/3G/ASI Serial Digital Interface (SDI), XAUI and Reduced XAUI (RXAUI), HiGig/HiGig+, SATA/Serial Attached SCSI (SAS), GPON, SerialLite II, Fiber Channel, SONET/SDH, Interlaken, Serial Data Converter (JESD204), SFI-5, and a host of others [7]. All the latter standards link modern high-speed and density devices either within the realm of a single board (chip-to-chip), in a multi-board design within a single machine, or in a machine-to-machine (M2M) communication scenario. While designing chip-to-chip interconnects on a single PCB, high-speed interconnect lines have strict impedance requirements to meet, which are listed in Table 1.

Even though there are multiple ways of calculating and analyzing [8] tracks and their impedances, including computer-aided design (CAD) electromagnetic simulations (EM), precise software impedance calculators and simplified equations, passive and active components with their own land-pads—usually different to the track width—are an inherent part of these interconnects. The designer has multiple degrees of freedom while shaping tracks to meet a certain impedance but is unable to heavily change the land-pad dimensions of the active and passive components in order to not corrupt the assembly quality while still meeting the target goal. As a result, each pad becomes a discontinuity and introduces negative effects to the transmitted signal. Thus, these pads, which in a controlled impedance chain appear as discontinuities, undergo impedance compensation techniques such as reference plane cut-out.

### 1.1. Motivation

The reference plane cut-out compensation technique, also sometimes referred to as compensation using a defected ground structure (DGS), has been a topic of research in multiple papers that cover different case–studies, such as SMA connector pads, track bends, etc., [9,10,11,12]. The main principle behind reference plane cut-out compensation is altering the per-length-capacitance and per-length-inductance of a discontinuity in a controlled impedance scenario by varying the depth of the reference (ground) plane in a multi-layer PCB.

Despite the fact that reference plane cut-outs help to mitigate the difference between track and pad impedances, the shift in impedance with each introduced cut-out is discrete and the step size depends on the number of available electrical layers, the thickness of each laminate, and the properties of the dielectric material. In some cases, a single reference plane cut-out is not enough to achieve the target impedance, but a successive cut-out on the next reference plane overshoots the result [11]. Therefore, the discontinuity impedance after the first compensation step is closer to Z_0_ than that without, but is still not enough, and the application of a second cut-out might even worsen the achieved result. The shapes of a cut-out under the discontinuity pad can be the same as the pad itself, or they can be different. The latter has been reported in various papers and design notes [13,14,15,16,17,18], but they are only single-case studies addressing passive surface-mount device (SMD) components such as 0201 or 0402 package size capacitors. Even though high-bandwidth digital applications usually employ small-size components, connectors, and other ICs in the chain, they still pose a challenge. Moreover, the analog RF and microwave realm does not utilize such large bandwidths compared to that of digital protocols, but correct chain impedance is critical for the highest power transfer. To the best of the authors’ knowledge, a generalized and quantified approach of applying reference plane cut-out compensation to various track-to-discontinuity ratios, where the shape of the cut-out differs from the initial size of the discontinuity, has not been covered. A generalized optimal cut-out size estimation could improve the quality multi-layer PCB design and provide an intuitive way of applying the latter technique without the aid of costly or time-consuming CAD solutions. Thus, a hypothesis is proposed, which is described by a mathematical model as well as proven by CAD simulations and measurement results. The proposed original approach of estimating an optimal cut-out width in dense designs can be used for solving practical engineering layout and signal integrity issues.

### 1.2. Estimating Practical Design Scenarios

In order to systematically approach the problem, the typical track-to-discontinuity ratios found in practical PCB designs must be established. Table 1 contains the typical multi-layer PCB design parameter ranges, such as laminate permittivity, laminate and copper thicknesses, as well as minimal distance between tracks in a differential pair. In order to avoid ambiguity in the terminology, a multi-layer PCB in this paper is considered to be a four or more copper layer one with a total thickness of around 1.6 mm, which is standard to the industry. Thicker laminate materials with higher dielectric permittivity also exist, but they are not considered in this paper—although the same analysis principle discussed in this paper would apply. Table 1 also holds the widths of the internal or external layer copper tracks, which are within a manufacturable set of parameter ranges (dielectric constant, laminate and copper thicknesses, as well as differential pair distance) with the target impedance for each of the given protocols, along with the typical SMD component pads. The integrated circuit package pad widths fall into the range of the latter, and thus, are standard passive.

The SMD components ranging from 0201 to 1206 in the imperial coding system are used as a reference for comparison. The following categories and design scenarios can be distinguished based on the results presented in Table 1:

1. Low-power, high-speed, high-density: 0201 size SMD components connected via smallest width tracks ([0.075 … 0.2] mm) can contain track-to-discontinuity ratio from 1:1.75 to 1:5;
2. Medium-power, medium-density: 0402, 0603 size SMD components connected via medium width tracks ([0.2 … 0.4] mm) can contain track-to-discontinuity ratio from 1:1.5 to 1:5;
3. High-power, medium/low-density: 0805 and larger size SMD components connected via larger width tracks ([0.4 … 0.71] mm) can contain track-to-discontinuity ratio from 1:2 to 1:5.

Thus, 1:1.75, 1:2.5, and 1:5.0 track-to-discontinuity ratios were selected in order to resemble most practical design scenarios. The smallest 1:1.5 ratio was not included due to the fact that discontinuities with such a ratio could be compensated only in a standard thickness multilayer PCBs with a large layer count hence were the thinnest laminates available. The results would neither be conclusive nor obvious in a 6-layer board, which is presented in this paper.

The paper is organized as follows: introduction and motivation for this research are followed by Section 2 with a theoretical analysis of the reference plane cut-out shapes and their impact on the discontinuity impedance. Section 3 holds the simulation and measurement results based on the typical track-to-discontinuity ratio analysis results. The results presented in this paper are then summarized in Section 4, with references provided afterwards.

## 2. Theoretical Evaluation

**Figure 1:**
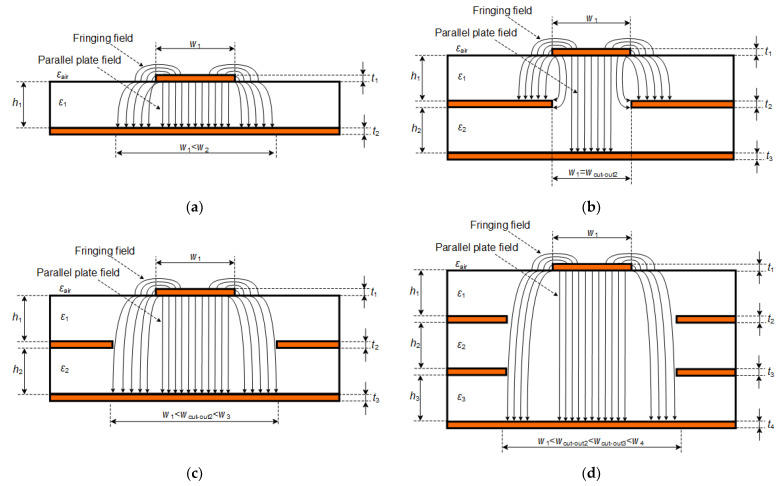
Microstrip line structures: (**a**) general; (**b**) with a cut-out whose width is equal to the width of the discontinuity pad; (**c**) with a cut-out whose width is much larger than the width of the discontinuity pad; (**d**) configuration with multiple cut-outs.

The general structure of a microstrip line contains a thin, flat conductor of width (*w*_1_) and thickness (*t*_1_), separated from a ground plane by a dielectric substrate of height (*h*_1_) and dielectric permittivity (*ε*_1_), as shown in Figure 1a. Theoretical analysis of the electromagnetic field is complicated by the fact that the microstrip line is surrounded by an inhomogeneous medium. Figure 1a shows a typical electric field pattern of this EM field. When the microstrip line geometry ratio is *w*_1_/*h*_1_ >> 1 and *ε*_1_ >> 1 (most common case of PCB structures), the electric field lines concentrate mostly in the substrate and some parts of the field lines exist through the air. Therefore, the propagating mode along the microstrip is not purely transverse electromagnetic (TEM) but quasi-TEM, consisting of fringing and parallel plate fields. It should also be noted that the fringing/edge electric field lines make the microstrip line look wider electrically than its physical dimensions. Furthermore, this has a significant effect on the total capacitance and, as a result, on the characteristic impedance of the microstrip line.

It is well known that the effects of inductance (*L*) and capacitance (*C*) have to be included in high-frequency circuit analysis. They make up the characteristic impedance parameter for a microstrip line, which can be determined as follows [19]:

(1) $$Z_{0}=\sqrt{\frac{R+j\omega L}{G+j\omega C}}={\left.\sqrt{\frac{L}{C}}\right|}_{R=0;G=0},$$

where *R* is the series resistance per unit length (Ω/m); *L* is the series inductance (H/m); *G* is the shunt conductance (Ʊ/m); *C* is the shunt capacitance (F/m). For an ideal, lossless line, which is considered in this simplified analysis, the parasitic series resistance *R* and parasitic parallel conductance *G* are equal to zero.

Clearly, adjusting this mathematical expression to be suitable for an ideal, lossless line shows that the characteristic impedance *Z*_0_ can be obtained by manipulating the per-length inductance or per-length capacitance of a microstrip. Sufficiently accurate mathematical methods and formulas for calculating *L* and *C* of a microstrip line can be found in various sources [20,21]. However, the *C* expression in these mathematical methods describes the total capacitance of a microstrip line consisting of both the parallel plate and the fringing capacitances, which are generated by the parallel plate and fringing components of the electric field pattern, respectively. Figure 1a also shows that the width of the electric field pattern (*w*_2_) on the ground plane is much larger than the width of the upper conductor (*w*_1_). Thus, knowing this width in the multilayer PCB structures, it would be possible to make a cut-out in the ground plane below, reduce the capacitance of the microstrip line, and thereby increase the characteristic impedance. However, determining this width accurately mathematically is problematic.

As mentioned in our earlier literature reviews [11,12], the most common design strategy is to make a cut-out the same width as the discontinuity, although such a window may be too small to minimize capacitance as much as possible. This is very clearly shown in Figure 1b, where the structure contains two dielectrics and three metal layers. In this stack-up/layer system, a cut-out with the same width (*w*_1_ = *w*_cut-out2_) as the microstrip line is created in the second layer, which is a reference (ground) plane. Similar simulation results are presented in [22], although they are shown as a case study for a 0402-package capacitor. However, this cut-out may be too small to minimize capacitance, as only the parallel plate capacitance component is reduced and the fringing one remains the same. Therefore, in order to minimize the total capacitance, the cut-out must be significantly larger than the width of the microstrip line (*w*_1_ < *w*_cut-out2_ < *w*_3_), as shown in Figure 1c.

In high-speed, high-density multilayer PCBs, impedance can be compensated for by adding a cut-out in multiple other layers as well. Such an example is given in Figure 1d. This allows a further increase in the characteristic impedance and possibly makes it closer to the target 50 Ω line impedance. However, even in these cases, the above cut-out must be close to or larger than the lowest cut-out in order to not form fringing capacitances in the respective layers and to maximally compensate for the characteristic impedance.

A simplified mathematical analysis solving for the reduction of the fringing capacitance involves having a per-unit-length capacitance model of choice, ex. [21]:

(2) $$C=\left\{\begin{array}{cc} \frac{\varepsilon_{eff}}{60v_{0}\ln\left(\frac{8h_{1}}{w_{1}}+\frac{w_{1}}{4h_{1}}\right)} & ,\frac{w_{1}}{h_{1}}\leq 1\\ \frac{\varepsilon_{eff}}{120\pi v_{0}}{\left(\frac{w_{1}}{h_{1}}+1.393+0.667\ln\left(\frac{w_{1}}{h_{1}}+1.444\right)\right)}^{} & ,\frac{w_{1}}{h_{1}}>1 \end{array}\right.,$$

where *ε_eff_* is the effective relative permittivity by Hammerstad and Bekkadal, *w*_1_ is the width of the microstrip and *h*_1_ is the thickness of the laminate, and the speed of light in a vacuum is denoted as *v*_0_ ≈ 3 × 10^8^ m/s. The latter capacitance expression describes the total *C* of a microstrip line consisting of both the parallel plate and the fringing capacitances generated by the parallel plate and fringing components of the electric field pattern, respectively.

The magnetic flux around the discontinuity with a per-length inductance is not taken into account, as capacitance is dominating in this segment and affecting the shift in impedance. The per-unit-length inductance of a microstrip line section was calculated according to [20,21]:

(3) $$L=\left\{\begin{array}{cc} \frac{60}{v_{0}}\ln\left(\frac{8h_{1}}{w_{1}}+\frac{w_{1}}{4h_{1}}\right) & ,\frac{w_{1}}{h_{1}}\leq 1\\ \frac{120\pi }{v_{0}}{\left(\frac{w_{1}}{h_{1}}+1.393+0.667\ln\left(\frac{w_{1}}{h_{1}}+1.444\right)\right)}^{-1} & ,\frac{w_{1}}{h_{1}}\geq 1 \end{array}\right.,$$

A hypothesis that suggests that the capacitance of a microstrip line discontinuity is compensated by cut-outs in multiple PCB layers can be assumed as the capacitance of a purely parallel type, leading to an optimal cut-out width and the impedance of the compensated discontinuity estimation with a given stack-up.

In order to calculate *w*_3_ in Figure 1c, and assuming that the capacitance of the microstrip line is of the purely parallel plate type, the expression of this width would be as follows:

(4) $$w_{3}=\frac{C\times (h_{1}+h_{2})}{\varepsilon_{0}\varepsilon_{eff}},$$

where *ε*_0_ = 8.854 × 10^−12^ F/m is the vacuum permittivity. Cut-out *w*_2_ in Figure 1c should be the same size as the estimated *w*_3_ or larger. Estimating the latter width for any multilayer PCB stack-up would lead to a possibility of making a cut-out in the reference plane of that particular size and optimally reducing the capacitance of the microstrip line and, thereby, increasing the characteristic impedance.

**Figure 2:**
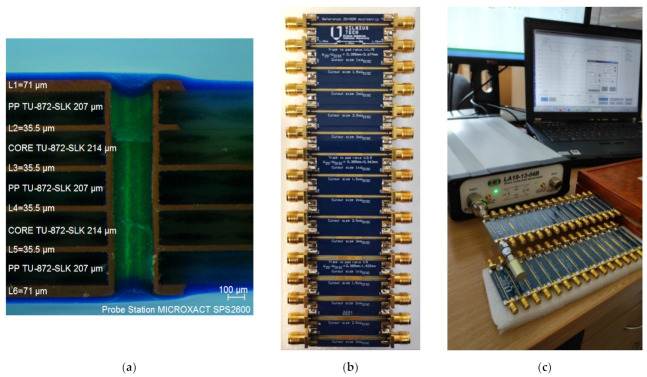
Manufactured 6-layer PCB with DUT structures: (**a**) optical microscopy evaluation; (**b**) top view; (**c**) measurement test-bench with LA19-1304B VNA.

**Figure 3:**
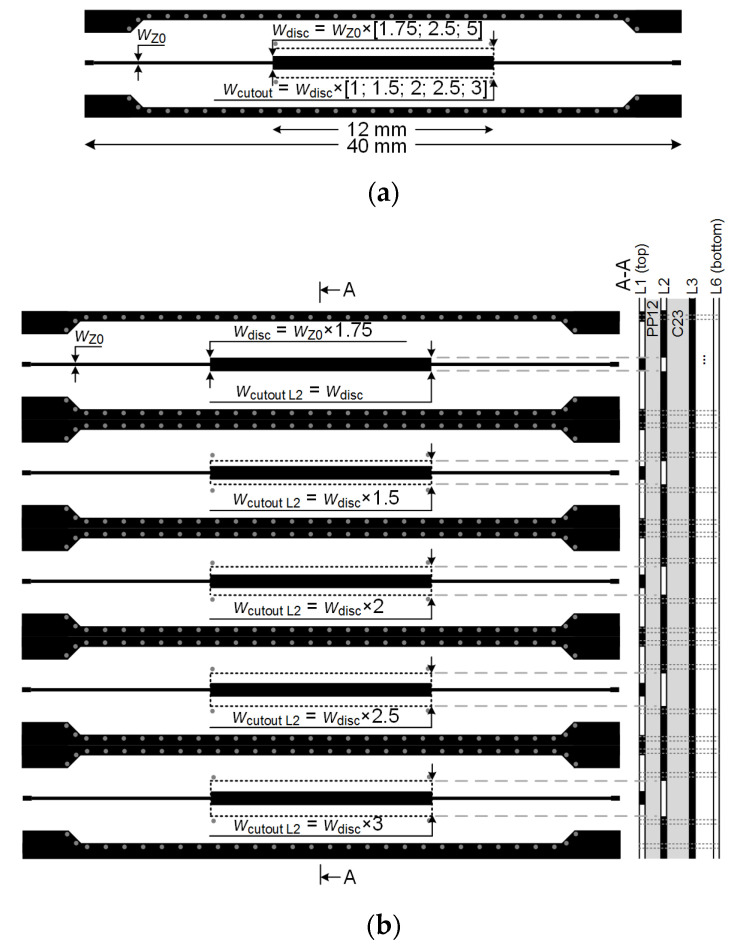

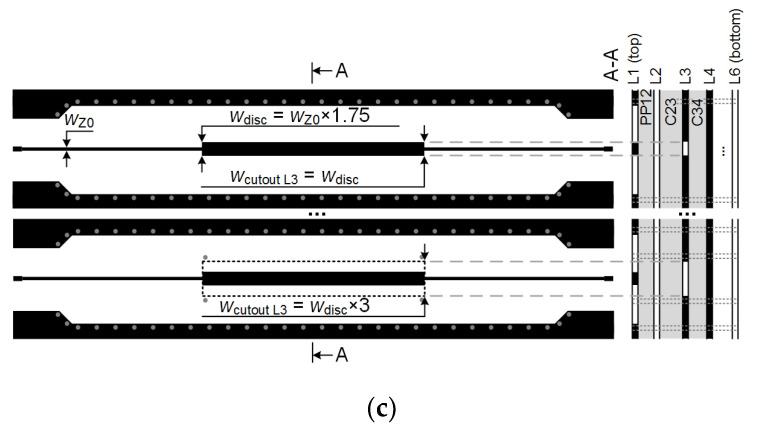
DUT structure description: (**a**) simplified description of the ratio between track, discontinuity, and cut-out; (**b**) detailed board top and side views with a track-to-discontinuity ratio of 1:1.75 and a single cut-out; (**c**) detailed board top and side views with a track-to-discontinuity ratio of 1:1.75 and a two-layer cut-out.

By applying the parameters of the designed PCB stack-up (parameters discussed in Section 3 of this paper and presented in Figure 2a) and the widths *w*_1_ of each DUT discontinuity pad (0.647 mm, 0.963 mm, 1.925 mm) in Equations (2) and (4), the theoretical optimal cut-out and discontinuity width ratios are found. For example, for a *w*_1_ = 0.647 mm discontinuity pad (track-to-discontinuity ratio of 1:1.75, as shown in Figure 3b) and a total laminate height including L2 copper thickness of 457 μm, the discontinuity capacitance *C* is calculated according to Equation (2) condition *w*_1_/*h*_1_ > 1 and is equal to 1.09 pF. The latter and the named total laminate height, as well as the effective relative permittivity for this structure (according to Hammerstad and Bekkadal model *ε*_eff_ ≈ 2.92), provides an optimal cut-out width of *w*_3_ ≈ 1.6 mm according to Equation (4). The latter ratios should provide the maximum achievable characteristic impedance in each case and are the following: for a discontinuity-to-microstrip line ratio of *w*_disc_/*w*_Z0_ = 1.75, the optimal cut-out and discontinuity ratio is found to be *w*_cut-out_/*w*_disc_ = 2.475; for *w*_disc_/*w*_Z0_ = 2.5, the ratio is *w*_cut-out_/*w*_disc_ = 2.059; and for *w*_disc_/*w*_Z0_ = 5.0, the ratio is *w*_cut-out_/*w*_disc_ = 1.603. Considering all of these width ratios, the capacitance in the impedance Equation (1) is found according to a purely parallel type of capacitance in Equation (4), whereas the inductance is calculated according to Equation (3). Thus, the maximum achievable impedances that apply a single cut-out are equal to Z_1.75_ = 62.8 Ω, Z_2.5_ = 49.9 Ω, and Z_5.0_ = 31.2 Ω, respectively. The latter theoretical estimation is verified via CAD simulations and practical measurements.

## 3. Simulation and Measurement Results

To test the proposed method described above, three types of DUT structures with different track-to-discontinuity ratios were selected according to Table 1: 1:1.75, 1:2.5, and 1:5.0. Each ratio indicates the ratio of the signal track to the discontinuity pad width. For example, with a signal track width of 0.385 mm (this width corresponds to the calculated 50 Ω impedance) and the pad width of 0.647 mm, this ratio is 1.75. For each of these ratios, five cut-outs of different sizes were formed in the reference inner-plane layer: 100%, 150%, 200%, 250%, and 300%. The latter DUT structure summary is presented in Figure 3. Figure 3a presents a generalized summary of the ratios used in the DUT measurement board, while Figure 3b,c showcase how the top PCB view corresponds to the internal stack-up when a single-layer or a two-layer cut-out compensation is applied. The latter figure only shows the detailed summary for different discontinuity-to-cut-out ratios with a track-to-discontinuity ratio of 1:1.75. The same approach is applied but not shown in the case of the 1:2.5 and 1:5.0 track-to-discontinuity ratios. The mentioned percentages indicate the size of the cut-out relative to the size of the discontinuity pad. For example, the largest cut-out is three times larger than the discontinuity pad and corresponds to 300%. All these microstrip DUT structures with discontinuities and cut-outs were designed and implemented on a six-layer PCB, with a low-loss TU-872-SLK (*D*_f_ = 0.008, *ε*_r_ = 3.9, *f*_rated_~10 GHz) core and prepreg layers. The fabricated DUT structures are presented in Figure 2b. The thicknesses of both the metal and dielectric layers were evaluated in the final fabricated DUT board. However, to estimate the manufacturer’s fabrication accuracy and to obtain the most accurate simulation results possible, the cross-section of this PCB was analyzed and the structural dimensions were accurately measured. Figure 2a presents a microscopic image taken on the cut cross-section surface of the six-layer PCB. The prepared multilayer PCB cross-sectional sample was photographed and measured using an integrated circuit/PCB measurement probe station, MICROXACT SPS2600. The sample was cut using a diamond blade saw and mounted in epoxy. The evaluation of the cross-section showed that the thicknesses of the upper and lower copper (Cu) layers were about 71 μm (2 oz), while the thickness of the inner Cu layers was about 35.5 μm (1 oz). Meanwhile, the prepreg TU-872-SLK and core TU-872-SLK dielectric thicknesses were equal to 207 μm and 214 μm, respectively.

**Figure 4:**
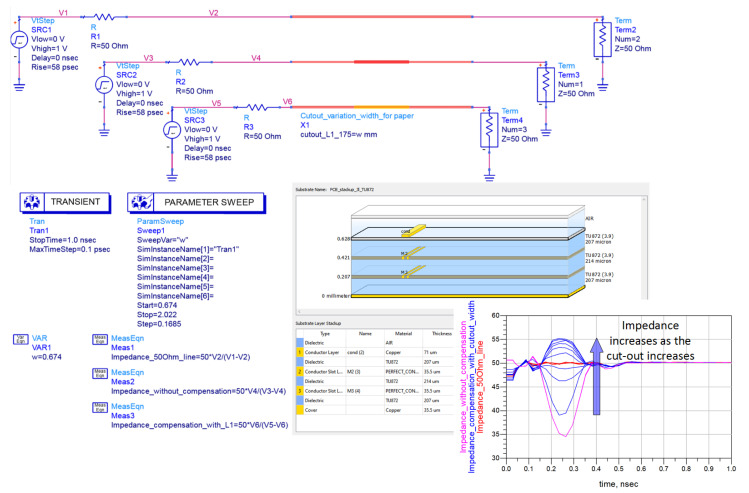
Keysight Agilent ADS test-bench schematic, stack-up and TDR response for a track-to-discontinuity ratio 1:1.75.

After an accurate estimation of the fabricated PCB layer thicknesses, a computer simulation of all DUT structures was performed. The EM simulator Keysight Agilent ADS, replicating the precise stack-up, was used for CAD simulations. The Keysight Agilent ADS test-bench schematic, stack-up, and simulation results for a track-to-discontinuity ratio of 1:1.75 is presented in Figure 4. Only three dielectric layers were defined in the stack-up during the test-bench setup, thereby simplifying the simulation process and taking into consideration that the stack-up is symmetric. A separate layout for each of the three simulated microstrip lines has been designed: a reference 50 Ω microstrip; a 50 Ω microstrip with a discontinuity and with no cut-out compensation; and a 50 Ω microstrip with a discontinuity and a width-varying cut-out for impedance compensation. The cut-out width is varied using a linear parametric analysis and ranges from 0.674 mm (corresponds to a discontinuity width of 100%) to 2.022 mm (corresponds to a discontinuity width of 300%) with a step of 0.1685 mm (a 25% of cut-out increase every step). The simulation results presented in Figure 4 contain a TDR response for the reference microstrip (red curve), a microstrip with a discontinuity and without compensation (pink curve), and a compensation cut-out width sweep (9 blue curves). It can be concluded that increasing the cut-out width from 0.674 mm to 2.022 mm increases the discontinuity impedance. Moreover, an overshoot above 50 Ω can be viewed and saturation can be spotted as each successive increase in width changes the impedance less than the previous. The value held as the impedance of the discontinuity is measured at the middle of the discontinuity length, which corresponds to an extremum on the TDR response.

**Figure 5:**
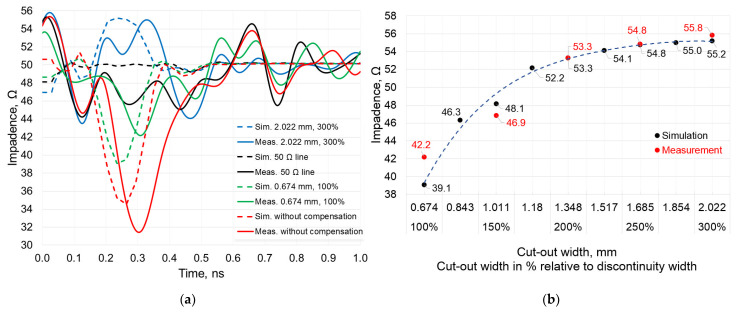
TDR simulation and measurement results for a track-to-discontinuity ratio of 1:1.75: (**a**) TDR response; (**b**) Impedance dependence on different cut-out sizes.

The time-domain reflectometry (TDR) measurements have been obtained using an open-short-load (OSL) calibrated 8.5 GHz bandwidth LA19-1304B vector network analyzer (VNA) with TDR capabilities. This VNA provides a rise time of around 58 ps, resulting in a minimal detectable discontinuity of around 5 mm, with all DUT discontinuities having a length of 12 mm. Each DUT segment was connected directly to the VNA, avoiding the use of additional cables, as shown in Figure 2c. The simulated and measured TDR responses of the track-to-discontinuity 1:1.75 ratio is presented in Figure 5a. For greater clarity, this graph shows only the intermediate measurement and simulation results: 50 Ω line, the microstrip with discontinuity and without compensation (red curves), and the discontinuities with 100% (green curves) and 300% (blue curves) cut-out widths. The maximum mismatch between the measurements and simulations is only about 10% and this mismatch decreases the closer the value is to 50 Ω. For example, in the absence of compensation, the simulation and measurement results are 34.7 Ω and 31.5 Ω, respectively. Meanwhile, at a 300% cut-out width, the simulation and measurement results are practically identical and equal to 55.2 Ω and 55.8 Ω, respectively. It should also be noted that the results of the simulations and measurements are shifted in time by 36 ps. This time shift is due to the additional length added to the discontinuity DUT structure by the SMA connectors, which has not been included in the CAD simulations.

The TDR simulation and measurement results show the impedance change over the DUT length. Although the horizontal axis is a time scale, it can be easily reverted to the distance travelled by the wave knowing the parameters of the PCB laminate. Summarizing Figure 5a, a large change in the impedance is seen at around 0.25 ns in simulation and at around 0.3 ns in the measurement TDR plots, both of which correspond to the reflection at the center of the discontinuity.

Although the curves themselves provide a lot of information in each case, the way they are presented in Figure 5a is not suitable for analyzing multiple simulation and measurement results. A clear visual trend of impedance change can’t be defined. Therefore, multiple TDR results have been transformed into the graph shown in Figure 5b, containing only the impedance values at the center of the discontinuity (the value seen at 0.25 ns or 0.3 ns, depending on whether it is a simulation or a measurement curve). This graph describes the dependence of impedance over different cut-out sizes at a 1:1.75 track-to-discontinuity ratio, providing ten simulation and five measurement points. Not all simulation points (cut-out sizes) were included into the final fabricated PCB to reduce the PCB dimensions and manufacturing costs. Analyzing the latter graph, the impedance transitions to the saturation state with a cut-out size of approximately 250% and its change with a further increase in the size of the cut-out is very small. For example, at a 100% cut-out size, the impedance was measured to be about 42.2 Ω. Impedances of 53.3 Ω and 55.8 Ω were measured at a 200% and 300% cut-out size, respectively. It should also be noted that the larger the size of the cut-out, the more precisely the simulations replicate the measurement results. The theoretical evaluation showed an optimum ratio between cut-out and discontinuity equal to *w*_cut-out_/*w*_disc_ = 2.475 times or around 248%.

**Figure 6:**
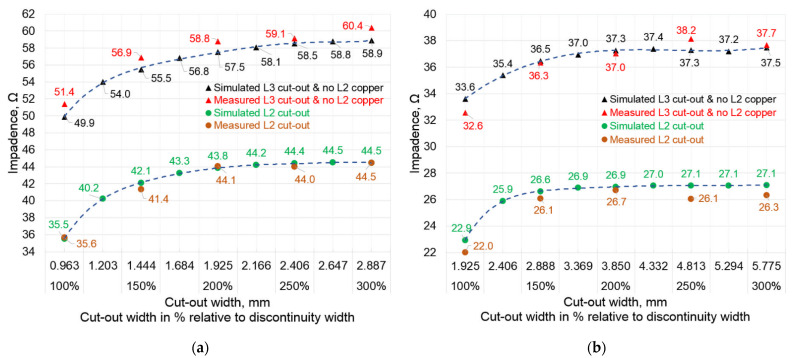
Impedance dependence on different cut-out sizes when cut-outs are made in the second L2 and third L3 inner PCB layers: (**a**) at 1:2.5 track-to-discontinuity; (**b**) at 1:5.0 track-to-discontinuity.

Practically identical dependences of the impedance change are obtained with cut-outs for other ratios (Figure 6a,b). For example, Figure 6a shows the dependence of impedance from the size of the cut-out at the *w*_cut-out_/*w*_disc_ = 2.5 ratio. It should be noted that this graph presents two curves—the bottom one is when cut-outs are made only in the second L2 and only in the third L3 inner PCB layers. Moreover, when the cut-outs were formed in the inner layer L3, there was no layer of copper in the upper L2 layer for that particular structure (Figure 3c). These impedance characteristics also have a saturation region from which the increase in impedance with increasing cut-outs is very small and insignificant. The starting point of the saturation region could also be considered when the cut-out is between 200% and 250%. The theoretical evaluation showed an optimum ratio of *w*_cut-out_/*w*_disc_ = 2.059 or almost 206%. In the case of cut-outs in the L3 inner PCB layer (Figure 6a), the impedance settles at the (59–60) Ω limit and reaches overcompensation, exceeding the target 50 Ω. Meanwhile, with the cut-out only in the L2 layer, the impedance settles at the (44–45) Ω limit. In the case of the 1:5.0 ratio (Figure 6b), the saturation region is observed at a slightly smaller cut-out size range of 150% to 200%. The impedance settles at (37–38) Ω and at (26–27) Ω, when cut-outs are in the L3 and L2 inner layers, respectively. The theoretical evaluation showed an optimum ratio of *w*_cut-out_/*w*_disc_ = 1.603 or almost 160%.

## 4. Conclusions

An original approach of estimating an optimal cut-out width in dense designs has been proposed and can be easily implemented for solving practical engineering layout and signal integrity issues. To test the proposed method using reference plane cut-outs, three types of discontinuity DUT structures with different ratios (1:1.75, 1:2.5, and 1:5.0) were designed, simulated, fabricated, and measured. Such discontinuity DUT structure ratios are chosen, given the widths of the base packages of the components currently common in the electronics industry and the typical track widths with controlled impedance requirements. A theoretical method of estimating the optimal cut-out size has been presented and evaluated through simulation and measurements. The measurements and simulations revealed that the compensated impedance saturation occurs at (150–250%) cut-out widths. For smaller track-to-discontinuity ratios (ex. 1:1.75), the optimal cut-out would be in the upper part of the mentioned range (>200%), and for larger ratios (ex. 1:5.0), the lower part (≤200%) of the range would apply. Therefore, a conclusion can be made that for high-density, high-speed PCBs with a similar stack-up, a cut-out twice as large as the discontinuity pad would provide a maximum characteristic impedance compensation and get as close as possible to the 50 Ω line with the existing PCB structure. Implementing the proposed research method in differential pairs could be useful for the future extension of this topic.

## Figures and Tables

**Table 1:** A summary of modern analog and digital interface impedances and typical multilayer PCB track width ranges.

Interface	Impedance, (Ω)	Configuration	Standard Design Parameters Used in Multilayer PCB Manufacturing	Manufacturable Track Width Range with a Given Impedance, (mm)	Typical SMD Package (0201 to 1206) Component Footprint Pad Width Range, (mm)
Radio frequency (RF) chains	50	Single-ended	*ε*_r_ = [3 … 5]; *h*_core/prepreg_ = [50 … 300] μm; copper thickness [0.5 … 2] oz; minimal distance between differential tracks 0.075 mm;	[0.075 … 0.71]	[0.35 … 2]
	75				
	100	Differential			
	150				
PCIe [2]	[85 … 90]				
USB2.x, USB3.x, USB4.x [1]	85				
LVDS [3], Serial RapidIO [4], CPRI [5], HDMI and other proprietary high-speed digital standards [7]	100				
DDR [6]	[50 … 60]	Single-ended			
	[100 … 120]	Differential			

## Data Availability

Not applicable.
